# A Subtype-Specific Critical Period for Neurogenesis in the Postnatal Development of Mouse Olfactory Glomeruli

**DOI:** 10.1371/journal.pone.0048431

**Published:** 2012-11-01

**Authors:** Yasuko Kato, Naoko Kaneko, Masato Sawada, Keishi Ito, Sousuke Arakawa, Shingo Murakami, Kazunobu Sawamoto

**Affiliations:** 1 Department of Developmental and Regenerative Biology, Nagoya City University Graduate School of Medical Sciences, Nagoya, Aichi, Japan; 2 Dpartment of Neuro-otolaryngology, Nagoya City University Graduate School of Medical Sciences, Nagoya, Aichi, Japan; City of Hope National Medical Center and Beckman Research Institute, United States of America

## Abstract

Sensory input is essential for the normal development of sensory centers in the brain, such as the somatosensory, visual, auditory, and olfactory systems. Visual deprivation during a specific developmental stage, called the critical period, results in severe and irreversible functional impairments in the primary visual cortex. Olfactory deprivation in the early postnatal period also causes significant developmental defects in the olfactory bulb, the primary center for olfaction. Olfactory bulb interneurons are continuously generated from neural stem cells in the ventricular-subventricular zone, suggesting that the olfactory system has plasticity even in adulthood. Here, we investigated the effect of transient neonatal olfactory deprivation on the addition of interneurons to the glomerular layer of the adult mouse olfactory bulb. We found that the addition of one subtype of interneurons was persistently inhibited even after reopening the naris. BrdU pulse-chase experiments revealed that the neonatal olfactory deprivation predominantly affected an early phase in the maturation of this neuronal subtype in the olfactory bulb. Subjecting the mice to odor stimulation for 6 weeks after naris reopening resulted in significant recovery from the histological and functional defects caused by the olfactory deprivation. These results suggest that a subtype-specific critical period exists for olfactory bulb neurogenesis, but that this period is less strict and more plastic compared with the critical periods for other systems. This study provides new insights into the mechanisms of postnatal neurogenesis and a biological basis for the therapeutic effect of olfactory training.

## Introduction

Sensory input during a specific time window, referred to as the critical period, is essential for the normal development of sensory centers in the brain [1]–[5]. The role of sensory input in the development of the visual system has been extensively studied. A lack of visual input during the developmental period results in severely impaired visual function. Hemilateral visual deprivation disturbs the establishment of normal circuitry, mainly in the primary visual cortex, and leads to permanent impairment of the cortical responsiveness to inputs from the deprived eye [6]–[11]. A similar effect of sensory deprivation during the early postnatal stage is reported in the auditory and somatosensory systems [1], [3], [4].

In animals, olfaction provides critical information about the environment, including the availability of food and mates, and the presence of dangers such as predators and toxic agents. Olfactory sensory neurons reside in the olfactory epithelium and express odorant receptors on their soma. These neurons project their axons to the main olfactory bulb (OB), the primary center for olfaction, where they are organized into structures called glomeruli [12].Sensory deprivation by naris occlusion beginning in the early postnatal period leads to reduced volume of the ipsilateral OB, increased apoptosis, the activation of microglia and astrocytes, and alterations in the expression levels of factors involved in neuronal development and function [13]–[20], suggesting that a “critical period” exists in the development of the olfactory system.

In the OB, although the projection neurons are generated only during late embryonic stages, GABAergic interneurons are mainly generated after birth. These neurons arise from neural stem cells through intermediate neuronal progenitors that reside in the ventricular-subventricular zone (V-SVZ), located at the lateral walls of the lateral ventricles, and this neurogenesis persists throughout life [21]–[23]. The newly generated interneurons migrate tangentially for a long distance through a pathway called the rostral migratory stream (RMS), toward the OB [24], [25]. After reaching the OB, they migrate radially to their final destination and differentiate into mature neurons. Most of them differentiate into granule cells in the granule cell layer, while about 10–15% of them differentiate into periglomerular cells (PGCs) in the glomerular layer [25]. Thus, olfactory interneurons are continuously replaced even after the developmental period. These features suggest that the olfactory circuitry maintains greater plasticity than the visual system. However, the effect of sensory input during specific time windows on the development of the olfactory system remains largely unknown. To address this issue, the functional and histological development of the OB after the transient deprivation of olfactory input during the early postnatal period needs to be analyzed.

In this study, we investigated the effect of transient olfactory deprivation during the early postnatal stage on the PGCs, which are located at the surface of the OB and directly receive input from the olfactory sensory neurons. A previous genetic fate-mapping study demonstrated that a constant net addition of PGCs occurs after birth, while postnatal neurogenesis contributes to only a minor fraction of the granule cell population [26]. Therefore, it is possible that the development of a normal PGC composition is more sensitive to olfactory deprivation than that of other populations in the OB. PGCs are classified into several subtypes by their expression of non-overlapping chemical markers, including tyrosine hydroxylase (TH), calbindin (CB), and calretinin (CR) [27], although their functional differences are not well understood. Here we compared the neurogenesis and cellular composition of the glomerular layer in mice before and after the olfactory recovery from deprivation, using a removable nasal occlusion plug [28]. Transient naris occlusion from postnatal day (P) 5 to P19 persistently suppressed the addition of new CR-positive (+) PGCs, but not of TH+ and CB+ PGCs, and caused functional impairment in adulthood. Both PGC addition and function substantially recovered in mice treated with odor enrichment for 6 weeks after naris reopening.

## Materials and Methods

### Animals

C57BL/6J wild-type mice were purchased from SLC (Shizuoka, Japan) and housed at constant room temperature with a 12-hr light/dark cycle (8∶00 and 20∶00) and access to food and water *ad lib*. All the experiments on live animals were performed in accordance with the guidelines and regulations of Nagoya City University. The protocol was approved by the Institutional Animal Care and Use Committee of Nagoya City University (approval number H22M-10). All surgery was performed under sodium pentobarbital, isoflurane, or hypothermia anesthesia, and all efforts were made to minimize suffering.

### BrdU Labeling

Mice were injected with bromodeoxyuridine (BrdU, 50 mg/kg, Sigma, St. Louis, MO) in phosphate-buffered saline (PBS). To examine the effect of transient naris occlusion in the early postnatal period on OB neurogenesis, newly born cells were labeled with BrdU injected once, just before removal of the left nasal plug at P19. Cell proliferation in the V-SVZ and migration through the RMS into the OB were analyzed 1 h and 1 week after removal of the plug. The distribution, early differentiation, and maturation of the BrdU+ cells in the periglomerular layer were analyzed 2 weeks, 3 weeks, and 6 weeks later, respectively. To investigate whether transient naris occlusion persistently suppressed the addition of new CR+ PGCs after naris reopening, the newly born cells were labeled with BrdU injected once at P33 and analyzed 6 weeks later (P75). In this series of analyses, the contralateral (right) hemisphere of the brain was used as a control.

### Reversible Olfactory Deprivation

Reversible olfactory deprivation using a naris occlusion plug [28] was performed as described previously [29]. Briefly, P5 mice were anesthetized by hypothermia, and a 5-mm-long occlusion plug coated with petroleum jelly was inserted into the left nasal cavity. To reopen the naris at P19, mice were sedated by an injection of sodium pentobarbital (60 mg/kg), and the plug was removed with forceps. In the behavior experiments, an occlusion plug was inserted into the contralateral (right) side of the nasal cavity the day before the odor discrimination test was performed, to specifically evaluate the olfactory function of the ipsilateral OB.

To confirm that the nasal occlusion specifically affected PGCs in the ipsilateral OB, we compared the numbers of TH+, CB+, and CR+ PGCs in the right (contralateral) OB in the “reopened” group with those in non-treated intact mice of the same age. There were no significant differences in the numbers of any of the three kinds of PGCs between the two groups (Fig. S1), suggesting that the olfactory manipulation-induced change in the number of PGCs observed in the ipsilateral OB was due to the altered olfactory input to the OB rather than to other indirect effects of the occluded nasal cavity.

### Odor Enrichment

After removal of the naris occlusion plug at P19, the mice were housed in an odor-enriched environment for 42 days, essentially as described [30]. The mice were exposed daily to a different odor placed in a tea ball hanging in a standard breeding cage, which was covered with a filter cap. The following 21 natural odoriferous items were purchased in a local grocery store and used for odor enrichment: lavender, garlic, paprika, marjoram, curry, rosemary, nutmeg, thyme, basil leaves, cumin, cardamom, tarragon, whole cloves, chocolate, celery, anise, lemon, ginger, orange, banana, and coffee. These odors were presented in the above sequence and repeated. As a control group, some mice were housed under the same conditions without odoriferous items in a tea ball.

### Odor Discrimination Test

The odor discrimination experiment was performed as described previously [31] with modifications as follows. Before starting the experiment, mice were placed in the test cage for 5 min to become familiar with the experimental conditions. As a habituation and control odor, 50 µL of freshly prepared paprika solution (10^−4^) and sterilized water, respectively, were placed onto a filter paper (2×2 cm, Advantec No.2), respectively. The paprika-containing filter paper was placed on one side of the cage, and the sterile water-containing one was placed on the other. Each mouse was presented with the paprika and water 5 times, for 5 min each, with 15-min intervals. After the five successive paprika trials, the mouse was presented with cinnamon as a novel odor for 5 min on the sixth trial. The behavior of each mouse in the test cage was recorded with a video camera, and the total sniffing time during each 5-min trial (reaction time) was measured. “Sniffing” was defined as the mouse placing its nose within 1 cm of the filter paper [31]. The reaction times between the first (Hab1) and subsequent trials (Hab2, Hab3, Hab4, and Hab5) for the habituation task, and between the 5th (Hab5) and 6th trials for the discrimination task (Test) were compared in each animal. At the odor concentration used in this test (10^−4^), intact mice did not show any preference for paprika or cinnamon (data not shown). The “discrimination index” was defined as 1 − (Hab5/Test), as previously reported [32].

### Immunohistochemistry and Quantification Analysis

Mice were deeply anesthetized with isoflurane and the brains were fixed by transcardiac perfusion with 4% paraformaldehyde in 0.1 M phosphate buffer, postfixed overnight in the same fixative, and 50-µm-thick floating coronal sections were prepared using a vibratome, as reported previously [33], [34]. The sections were incubated for 40 min in 1% H_2_O_2_ in PBS, 30 min in blocking solution (10% normal donkey serum and 0.2% Triton-X 100 in PBS), overnight at 4°C with primary antibodies, and 2 h at room temperature with biotin- or Alexa-Fluor-conjugated secondary antibodies (1∶500, Invitrogen, Carlsbad, CA) in the same solution. The signals were amplified with biotin-conjugated antibodies and the Vectastain Elite ABC kit (Vector Laboratories, Burlingame, CA), and visualized using the TSA Fluorescence Systems (Perkin Elmer, Waltham, MA). For BrdU staining, the sections were pretreated with 2 N HCl for 40 min at 60°C before H_2_O_2_ incubation. The following primary antibodies were used: rat anti-BrdU antibody, 1∶100 (Abcam, Cambridge, MA); mouse anti-CB monoclonal antibody (IgG), 1∶3000 (Sigma, St. Louis, MO); rabbit anti-CB antibody, 1∶100 (Cell Signaling Technology, Danvers, MA); mouse anti-CR monoclonal antibody (IgG), 1∶3000 (Chemicon, Billerica, MA); goat anti-Dcx antibody, 1∶100 (Santa Cruz Biotechnology, Santa Cruz, CA); mouse anti-Mash1 monoclonal antibody (IgG), 1∶100 (Becton, Dickinson and Company, Franklin Lakes, NJ); and mouse anti-TH monoclonal antibody (IgG), 1∶1600 (Chemicon, Billerica, MA). Nuclei were stained with Hoechst 33342 (Sigma, St. Louis, MO).

To characterize the PGCs, the colocalization of signals in the glomerular layer was confirmed by scanning at 2-µm intervals. The scanning was performed using a confocal laser-scanning microscope, LSM5 Pascal (Carl Zeiss, Oberkochen, Germany), with a water-immersion objective (40×). All the double-positive cells in the glomerular layer were counted. To quantify the number of each PGC subtype, the cells in the glomerular layer were counted using a Stereo Investigator system (MBF Bioscience, Williston, VT), because they were densely packed in the OB. In the immunohistological analyses for double-positive and single-positive cells, the number of cells in every twelfth or sixth 50-µm-thick coronal section of the whole OB that contained the glomerular layer (at least 5 or 10 sections in total) was counted, then the total number was estimated by multiplying the number of counted cells by twelve or six, respectively.

To quantify the Mash1+ cells in the V-SVZ and the BrdU+ cells throughout the V-SVZ, RMS, and OB, images of every 6th section stained for Mash1 or BrdU, respectively, were acquired using a fluorescence microscope, BX51 (Olympus, Tokyo, Japan), and a CCD camera, DP71 (Olympus, Tokyo, Japan). Using the captured images, the number of BrdU+ cells was separately counted in 5 regions of the V-SVZ (medial, ventral, lateral, dorsal, and cortical), the RMS, and 4 regions of the OB (glomerular layer, external plexiform layer, granule cell layer, and core), and the number of Mash1+ cells was separately counted in the 5 regions in the V-SVZ, the RMS, and the entire OB. The number of BrdU+ and Mash1+ cells in each region was multiplied by six.

### Statistics

The data obtained in this study are presented as the mean ± SEM. The normal distribution and homogeneity of variance were checked, and comparisons between two groups were analyzed by the paired *t*-test, unpaired *t*-test or Welch’s *t*-test. For comparisons among multiple groups, the data were analyzed by one-way ANOVA, followed by the *post-hoc* Tukey-Kramer test. The data from the odor discrimination test were analyzed by repeated measures ANOVA followed by *post-hoc* pairwise comparisons (Fisher’s PLSD). Differences were regarded as statistically significant when *P*<0.05.

## Results

### Transient Olfactory Deprivation in the Early Postnatal Period Continuously Suppresses the Addition of New CR+ PGCs

**Figure 1:**
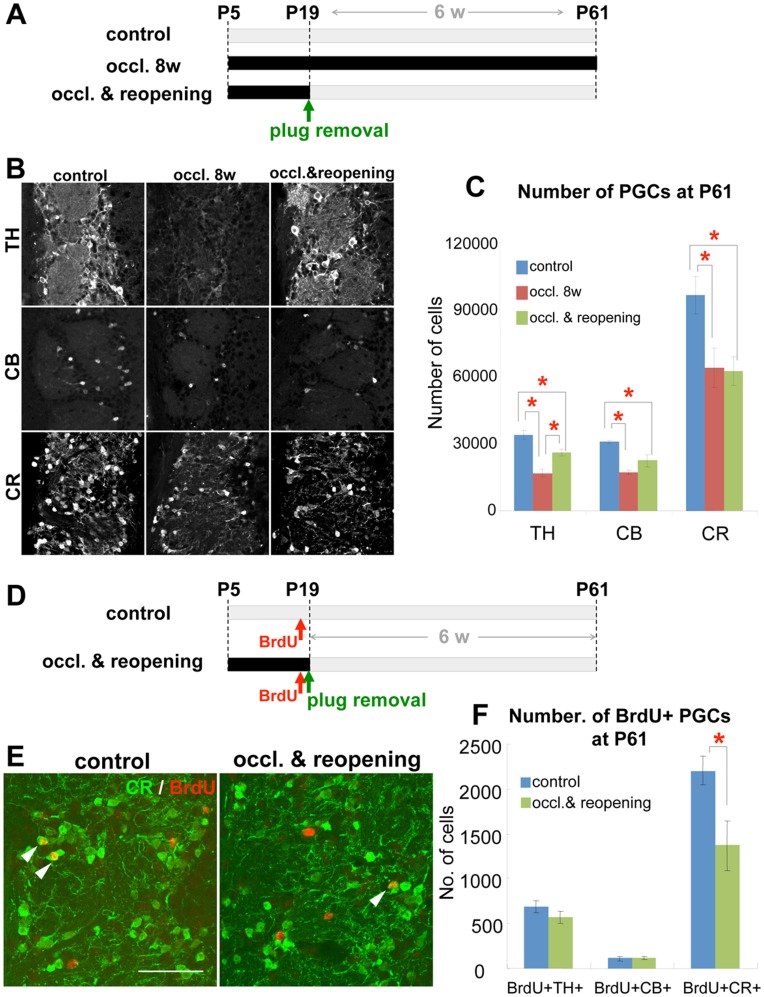
Transient neonatal olfactory deprivation causes a persistent decrease in the addition of CR+ PGCs. A : Experimental procedure. To block olfactory input, an occlusion plug was inserted into the left nasal cavity of P5 mice. The plug was kept in for 8 weeks (occl. 8w) or removed 2 weeks later at P19 (occl. & reopening). The contralateral OB of the occl. & reopening group was used as a control (control). The number of PGCs was quantified at P61. **B–C**: Effect of transient neonatal olfactory deprivation on PGC populations. Coronal sections of the glomerular layer of P61 mice of each group were immunostained for TH, CB, and CR (**B**). The numbers of all three types of cells were significantly decreased by the 8^th^ week of naris occlusion (**C**, red bars, *n* = 4) compared with the control group (**C**, blue bars, *n* = 5). The TH+ PGC population, but not the CB+ or CR+ population, was significantly larger in the reopening group (**C**, green bars, *n* = 5) compared with the continuous occlusion group (**C**, red bars). *P<0.05. **D**: Experimental procedure. Mice undergoing 2-week hemilateral naris occlusion from P5 were injected with BrdU just before naris reopening by nasal-plug removal at P19. The number of BrdU+ PGCs was quantified 6 weeks later, at P61. **E–F:** Effect of transient neonatal olfactory deprivation on the addition of PGCs. Coronal sections of the glomerular layer in the OB 6 weeks after BrdU injection were immunostained for BrdU and PGC markers TH, CB, and CR (*n* = 4). Arrowheads in **E** indicate BrdU+CR+ cells in the control (left) and occl. & reopening group (right). The number of BrdU-labeled CR+ PGCs, but not TH+ or CB+ PGCs, was significantly smaller in the occl. & reopening group (green bars) compared with the control, non-occluded group (blue bars) (**F**). *P<0.05. Scale bars: 50 µm.

To examine the effect of transient olfactory deprivation during the early postnatal period on the cellular composition of the periglomerular layer in adulthood, hemilateral naris occlusion was performed using a nasal plug at P5, and the naris was reopened 2 weeks later (at P19) (Fig. 1A). The 2-week naris occlusion caused a significant reduction in the volume of the granule cell layer and the glomerular layer (data not shown) and in the numbers of all three PGC subtypes, i.e., PGCs expressing TH, CB, or CR, at P19 (Fig. S2). Removal of the occlusion plug resulted in complete recovery of the volume of the OB layers (Fig. S3). The numbers of all the PGCs were still smaller than in the control; however, the ratios of the number of TH+ and CB+ PGCs in the ipsilateral OB to that in the contralateral (control) OB 6 weeks after naris reopening (P61) were significantly increased compared with the ratios at the end of the occlusion (P19) (Fig. S4). On the other hand, the number of CR+ PGCs did not show such a recovery 6 weeks after plug removal (at P61) (Fig. 1B, C, S4).

Under physiological conditions, CR+ PGCs are actively generated during the postnatal period, including adulthood [35]–[37]. Therefore, we hypothesized that the early postnatal olfactory deprivation suppressed the generation of CR+ PGCs and/or their tangential or radial migration, survival, and integration into the glomerular circuitry even after naris reopening. To test this possibility, newly born cells were labeled with BrdU injected just before removal of the nasal plug at P19. The number of BrdU+ cells co-expressing one of the PGC markers in the glomerular layer was counted 6 weeks later (at P61) (Fig. 1D). Interestingly, the number of BrdU+CR+ cells, but not of BrdU+TH+ cells or BrdU+CB+ cells, in the adult glomerular layer was significantly reduced by the early postnatal nasal occlusion (Fig. 1E, F). A similar subtype-specific reduction in CR+BrdU+ cells was observed when mice received a BrdU injection 2 weeks after naris reopening (at P33), and the cells were quantified 6 weeks later (at P75) (Fig. S5). These results indicate that transient olfactory deprivation in the early postnatal period persistently suppresses the addition of new CR+ PGCs to the OB.

Recent studies indicate that specific subtypes of olfactory interneurons are generated within specific locations of the V-SVZ and RMS [38], [39]. Since the CR+ PGCs are reported to be generated in the RMS and anterior and medial V-SVZ [39], we studied the effect of early postnatal olfactory deprivation on the number of Mash1+ neuronal progenitor cells in these regions 0 (P19) and 6 weeks (P61) after removal of the nasal plug. We did not find any difference in the number of Mash1+ cells in these regions (Fig. S6). We also quantified the number of BrdU+ cells in these regions 1 h after BrdU injection (at P19). There was no significant difference in the number of BrdU+ proliferating cells between the control and transiently deprived OB (Fig. S7A, B). Furthermore, we compared the distribution of BrdU-labeled interneurons one week after BrdU injection. There were no significant differences in the numbers of BrdU+ cells in any region of the RMS or OB between the control and occluded hemisphere one week after BrdU injection (Fig. S7C, D). These results suggest that the transient nasal occlusion during the early postnatal period did not alter cell proliferation or migration in the V-SVZ and RMS, similar to the results of our previous study in adult mice [29].

**Figure 2:**
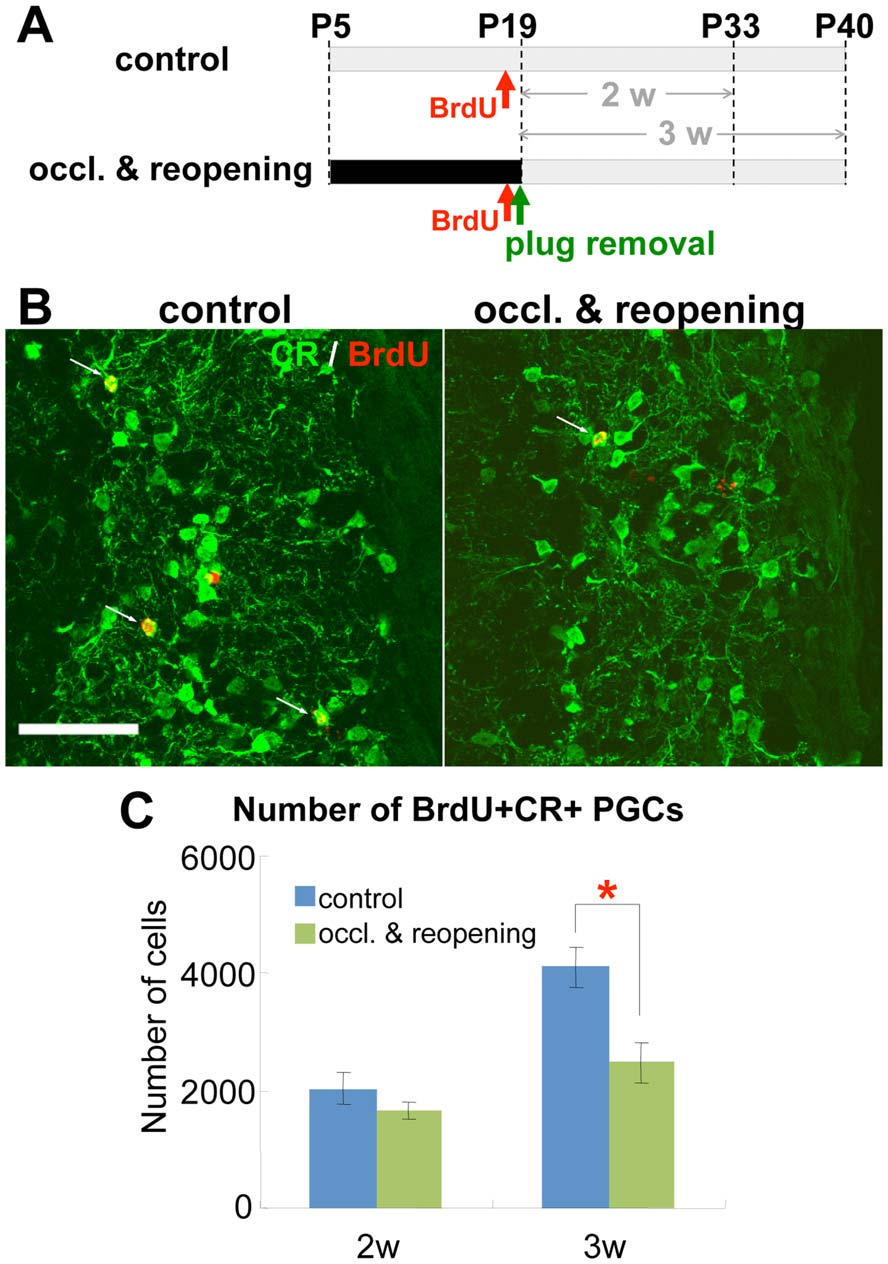
Transient neonatal olfactory deprivation affects the early differentiation phase of CR+ PGCs in the OB. A: Experimental procedure. Mice undergoing 2-week hemilateral naris occlusion (P5–P19) were injected with BrdU just before naris reopening by removal of the nasal plug, and the number of BrdU+CR+ PGCs in the glomerular layer was quantified 2 weeks (2 w) and 3 weeks (3 w) later, at P33 and P40. **B–C**: Effect of transient neonatal olfactory deprivation on newly generated CR+ PGCs. Coronal sections of the glomerular layer in the OB 2 weeks (*n* = 3) and 3 weeks (*n* = 3) after BrdU injection were prepared and immunostained for BrdU (red) and CR (green) (**B**). Arrowheads in **B** indicate BrdU+CR+ cells. The number of BrdU+CR+ PGCs was significantly smaller in the occl. & reopening group (green bars) compared with the control, non-occluded group (blue bars) 3 weeks (P40), but not 2 weeks (P33), after BrdU injection. *P<0.05. Scale bar: 50 µm.

To investigate the time course of the decrease in CR+ cells, we quantified the number of BrdU+CR+ cells 2 and 3 weeks after the BrdU administration and plug removal (Fig. 2A). The number of BrdU+CR+ PGCs in the glomerular layer of the OB on the transiently occluded side was significantly decreased at 3 weeks (P40), but not at 2 weeks (P33), compared with the non-occluded side (Fig. 2B, C). The amount of reduction of BrdU+CR+ PGCs at 3 weeks (P40) (40.0±6.1%) (Fig. 2C) was similar to that at 6 weeks (P61) (38.6±9.7%) (Fig. 1F), suggesting that the decrease in the number of labeled CR+ cells, observed at P61 in mice with transient olfactory deprivation during their neonatal period (P5–19), resulted from defects in neuronal maturation events during the third week after their labeling. Taken together, these results show that early postnatal olfactory deprivation specifically disturbed the addition of CR+ PGCs, which might be involved in the long-lasting decrease of CR+ PGCs compared with the non-deprived OB, seen even after naris reopening.

### An Odor-enriched Environment Reverses the Reduction in CR+ Cells Caused by Early Postnatal Olfactory Deprivation

**Figure 3:**
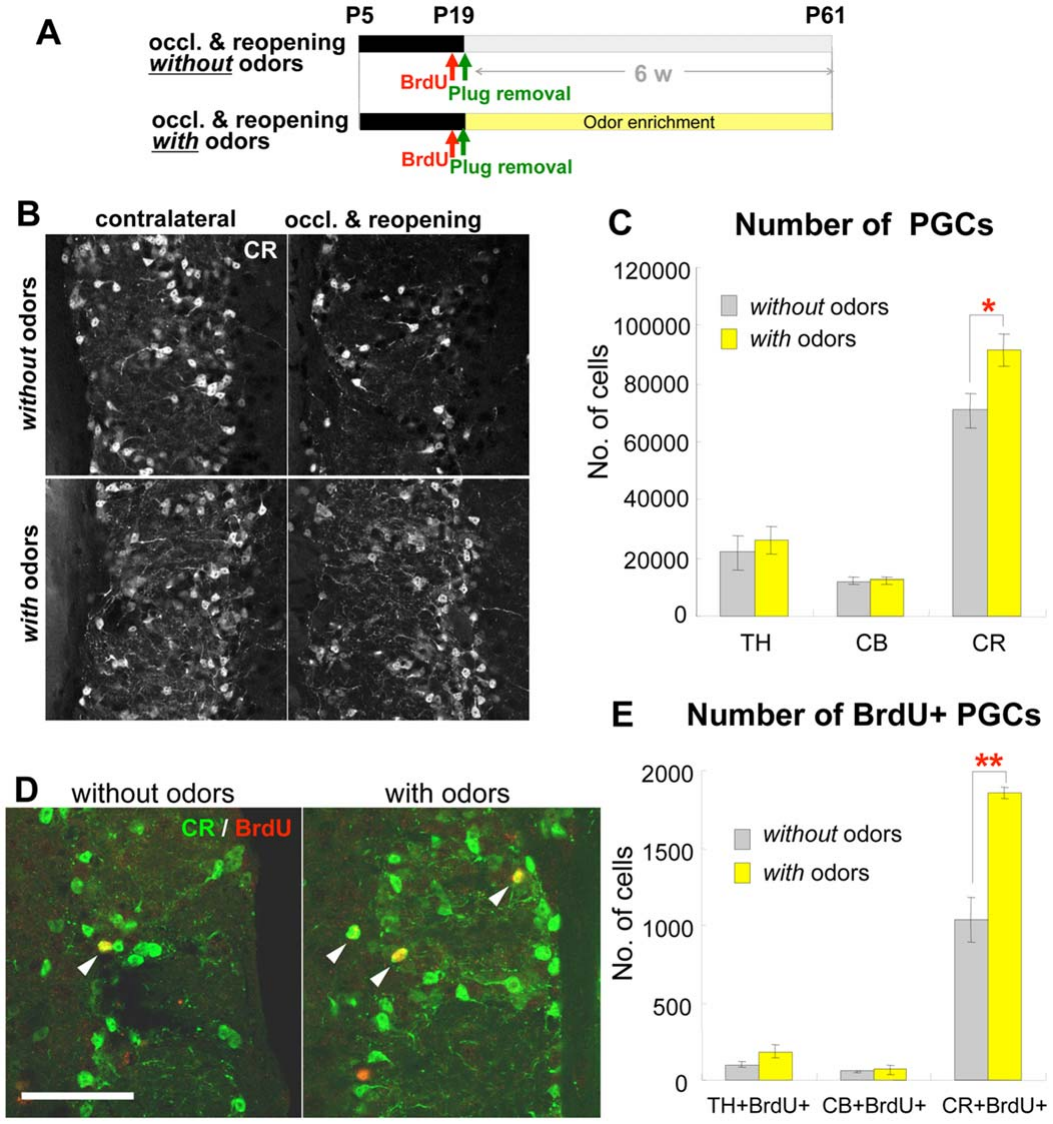
Odor stimulation recovers the reduction in CR+ PGCs caused by transient neonatal olfactory deprivation. A : Experimental procedure. Mice undergoing 2-week hemilateral naris occlusion from P5 were injected with BrdU just before naris reopening at P19 by removal of the nasal plug, then exposed to 21 different odorants that were changed every day for 6 consecutive weeks (*with* odors) or left in the normal breeding conditions without odor presentation (*without* odors). Histological analyses were performed at P61. **B–C**: Effect of odor stimulation on PGC populations. Coronal sections of the glomerular layer of P61 mice of each group, immunostained for CR (B). The number of CR+ PGCs, but not of TH+ or CB+ PGCs was significantly increased in mice exposed to odors (with odors, **B**, bottom panels; **C**, yellow bars, *n* = 5) compared with mice not exposed to presented odors (without odors, **B**, top panels; **C**, gray bars, *n* = 6). *P<0.05** D–E:** Effect of odor stimulation on the addition of PGCs. Coronal sections of the glomerular layer of P61 mice of each group, immunostained for BrdU (red) and CR (green) (**E).** Arrowheads in **E** indicate BrdU+CR+ cells. The number of BrdU+CR+ PGCs, but not of BrdU+TH+ or BrdU+CB+ PGCs, was significantly increased in mice exposed to odors (*with* odors, **D**, right panel; **E**, yellow bars, TH and CB: *n* = 3, CR: *n* = 4) compared with those in mice without odor presentation (*without* odors, **D**, left panel; **E**, gray bars, TH and CB: *n* = 3, CR: *n* = 4). **P<0.01 Scale bars: 50 µm.

Since odor stimulation promotes olfactory neurogenesis [30], [40]–[42], we tested whether the suppressed addition of CR+ cells in mice following early postnatal olfactory deprivation could be reactivated by exposure to various natural odors. After a two-week naris occlusion (P5–19), mice were injected with BrdU right before naris plug removal at P19, and then exposed for 6 weeks to a succession of natural odors, changed daily, that promote neurogenesis [30] (Fig. 3A). In the contralateral, non-occluded OB, there was no significant difference in the numbers of CR+ PGCs between the groups (without odor stimulation: 104,700±7,800 cells, *n* = 6; with odor stimulation: 110,400±5,700, *n* = 5, *P*>0.05), suggesting that odor stimulation alone does not induce CR expression. On the occluded side, the number of BrdU+CR+ cells was significantly higher in mice exposed to the odors compared with that in mice without odor stimulation (Fig. 3B, C). Moreover, the total number of CR+ PGCs, but not of TH+ or CB+ PGCs, was also increased by the odor enrichment (Fig. 3D, E). Thus, olfactory stimulation promoted the addition of new CR+ PGCs and recovery from the reduction in the CR+ PGC population caused by early postnatal olfactory deprivation.

### An Odor-enriched Environment Improves the Olfactory Function in Mice Exposed to Early Postnatal Olfactory Deprivation

Finally, we tested whether the exposure of mice to odor enrichment could also improve their olfactory function after early postnatal olfactory deprivation. To evaluate short-term odor memory and odor discrimination, we performed a habituation/dishabituation test [31], [43] on mice after neonatal olfactory deprivation followed by 6-week odor stimulation as described above (Fig. 3A). The day before the behavior test, an occlusion plug was inserted into the contralateral (right) naris, so the mice could perceive odors only through the ipsilateral (left) OB, which had been deprived of olfactory input for the 2 weeks after P5.

**Figure 4:**
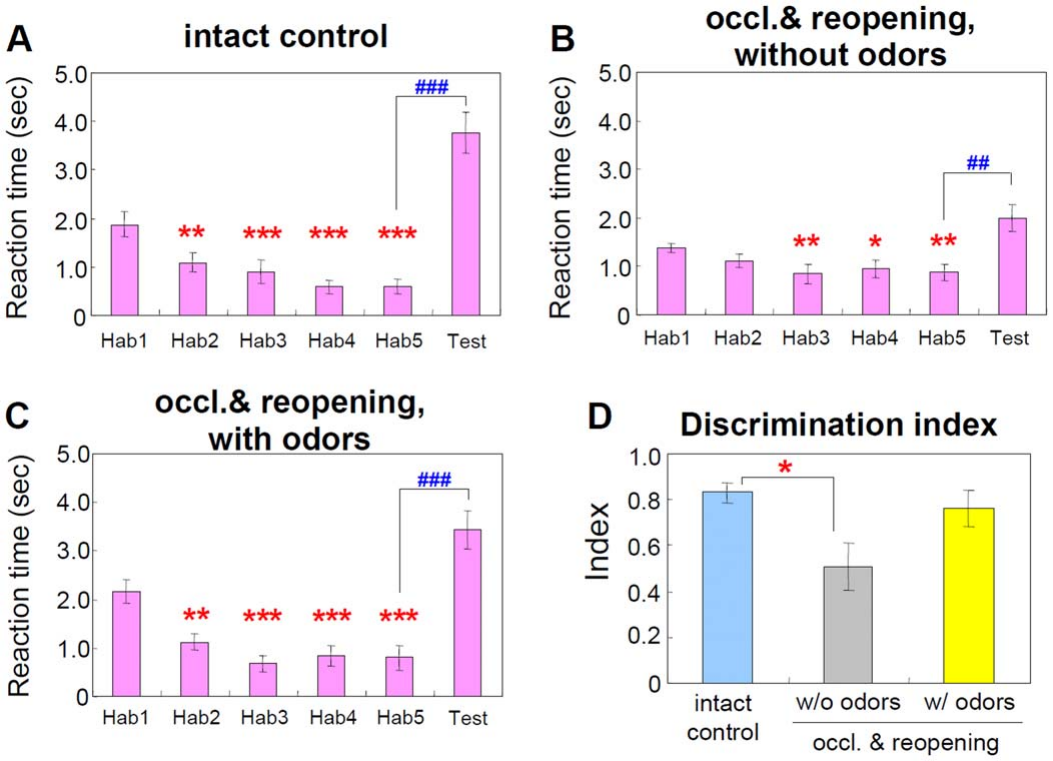
Odor stimulation improves olfactory behaviors in mice exposed to olfactory deprivation in the neonatal period. Odor habituation and discrimination test. In behavioral habituation to repeated presentation of a single odor (paprika), the reaction time to the habituation odor in the second (Hab2) and following 3 trials (Hab3–5) was significantly lower than in the first trial (Hab1) in the non-treated control mice (intact control, **A,** *n* = 14). The reaction time in mice without odor stimulation after naris reopening was also significantly lower in Hab3–5, but not in Hab2 compared with Hab1 (**B,** *n* = 11). The reaction time in mice treated with odors showed a similar pattern to that in the intact control group (**C,** *n* = 8). In the odor discrimination test, the response time to a novel odor (Test) was significantly longer than in the last habituation trial (Hab5) in all the groups. However, note that the discrimination index (**D**) was significantly lower in the group without odor stimulation after naris reopening (without odors, gray bar), but not in the odor stimulation group (with odors, yellow bar), compared with the control group (intact control, pale blue bar). *P<0.05, **P<0.01, ***P<0.001, ^##^ *P*<0.01, ^###^ *P*<0.001.

In habituation trials (Fig. 4A–C), the same odor (paprika) was presented 5 times for 5 minutes with 15-minute intervals. Mice in the control group (intact control), which did not receive any treatment, showed a significant decrease in investigation time during habituation trials 2 to 5 (Hab2–5), compared to the first trial (Hab1) (Fig. 4A). The mice that did not receive odor stimulation after naris reopening showed a significant decrease in investigation time only after the second trial (Hab3–5) (Fig. 4B). On the other hand, the mice treated with odor stimulation showed a normal habituation, similar to the mice in the control group (Fig. 4C).

We next performed an odor discrimination test (Fig. 4A–C, Hab5 and Test), in which we compared the investigation time for a novel odor (cinnamon, Test) to that in the last trial for the habituation odor (Hab5) [31]. Although the investigation time for the novel odor was significantly longer compared with the last habituation odor in all three groups, the discrimination index, which allowed us to compare the performance independent of the overall variation in investigation time [32], was significantly lower in the untreated reopened group, but not in the odor-enriched reopened group, compared with the control group (Fig. 4D). These results suggest that transient neonatal olfactory deprivation impairs olfactory functions in adult mice, which can be recovered by subsequent exposure to an odor-enriched environment.

## Discussion

### Early Postnatal Olfactory Deprivation Causes the Persistent Reduction of CR+ PGCs

The addition of new interneurons in the OB is regulated by olfactory input. Olfactory deprivation leads to apoptosis of the V-SVZ-derived new neurons during maturation in the granule cell layer and glomerular layer [44]–[46]. In adulthood, olfactory deprivation causes suppression of TH expression [47] and a decrease in the number of new TH+ PGCs [29], but does not affect the CR+ or CB+ PGC population [29], [41], [48]. On the other hand, olfactory deprivation for a few weeks in the early developmental stage severely diminishes the volume of the OB layers and the absolute numbers of CB+ and CR+ PGCs (Fig. S2) [13], [49], indicating that the development of the OB is highly dependent on olfactory input. Within 10 days after naris reopening following neonatal olfactory deprivation, apoptotic cell death decreases to the normal level [17], followed by recovery of the volumes of the OB layers including the glomerular layer [28] (Fig. S3), suggesting that normal OB cytoarchitecture and olfactory function can develop after olfactory recovery.

Using an unbiased stereological method to count the total number of PGCs, we found that, while the numbers of TH+, CB+, and CR+ PGCs were similarly decreased by neonatal naris occlusion (Fig. S2), only the CR+ PGC population showed no recovery 6 weeks after naris reopening (Fig. S4), remaining at a level similar to that in the OB with a continuously occluded naris (Fig. 1C). Precise examination of the added PGCs revealed that the number of new CR+ PGCs, but not of the TH+ or CB+ ones, was persistently lower in the OB of the previously occluded naris compared with the non-occluded naris. Both TH+ and CR+ PGCs are continuously added to the OB under physiological conditions throughout life [35]–[37]. Therefore, it was possible that the on-going decrease in CR+ PGCs was due to a persistent reduction in the addition of new CR+ PGCs after transient neonatal olfactory deprivation.

Our BrdU pulse-chase experiments suggested possible steps in the addition of new CR+ PGCs to the glomerular layer, which can be affected by transient olfactory deprivation. New neurons are generated in the V-SVZ and first migrate anteriorly in the RMS and then radially in the OB during the first week. Some of these cells reach the glomerular layer in the second week, where their survival and differentiation are regulated by sensory input during the third and fourth weeks [44]. We found that the numbers of proliferating cells and neuronal progenitors in the medial V-SVZ and RMS, where CR+ PGCs are predominantly produced, were not affected by olfactory deprivation (Fig. S6, S7A, B), suggesting that the production and rostral migration of new neurons did not depend on olfactory input, consistent with previous reports [29], [30]. The BrdU+ cells at 1 week (Fig. S7C, D) and new CR+ PGCs at 2 weeks (Fig. 2) were distributed normally in the OB after BrdU labeling, suggesting that the radial migration was also not affected. However, the number of BrdU+CR+ PGCs was significantly decreased at 3 weeks, by about 40%, which was comparable to the reduction 6 weeks after BrdU injection (Fig. 2). These results suggest that the transient neonatal olfactory deprivation specifically affected new CR+ PGCs in the early phase of differentiation in the glomerular layer, rather than their generation, migration, or later differentiation phases. In contrast, the addition of CR+ PGCs in the adult OB is not affected by olfactory input [29], [41], [48], indicating that their dependency on sensory input is altered during OB development. Neonatal olfactory deprivation is reported to alter the expression levels of various genes and proteins involved in neuronal survival and/or maturation [14], [18]–[20], which might be involved in the molecular mechanisms underlying the sensory deprivation-induced perturbation in the early phase of CR+ PGC differentiation.

### Odor Stimulation Recovers Functional and Structural Impairments Caused by Transient Neonatal Olfactory Deprivation

Exposure to an odor-enriched environment and olfactory learning experience promotes the survival of new neurons in the OB [30], [40]–[42], [50] and improves olfactory function [30] in normal mice not subjected to olfactory deprivation. Olfactory stimulation also has a therapeutic effect in human patients with anosmia [51], although it has been unclear how odor exposure improves olfaction. We found that 6 weeks of odor stimulation significantly increased the addition of CR+ PGCs, but not of TH+ or CB+ ones, in mice after neonatal olfactory deprivation. In contrast, a previous study with intact mice reported that odor enrichment increased the number of new PGCs in a non cell-type-specific manner [41]. Although the mechanism of the cell-type-specific effect of this treatment remains unclear, it is likely to be a compensatory response to the persistent subtype-specific reduction in CR+ PGCs caused by the transient neonatal olfactory deprivation.

To investigate the effects of transient neonatal olfactory deprivation and subsequent odor enrichment on olfactory function in adulthood, we needed to exclude olfactory input through the contralateral OB during the olfaction tests. For this purpose, we occluded the contralateral naris with a nasal plug during the olfactory behavior test. Consequently, we could detect significant functional impairments in odor habituation and discrimination tasks through the previously occluded OB, which were improved by 6 weeks of odor stimulation. Since previous studies suggest that new interneurons are involved in the odor discrimination task [31], [40], [52], it is possible that the new CR+ PGCs that were increased by odor enrichment contributed to the functional improvement, at least in part. Neonatal olfactory deprivation also affects glial cells [16], [53] and primary relay neurons [54], [55] in the OB, which can also contribute to the recovery of olfactory function. Taken together, although olfactory deprivation in the early postnatal period caused continuous defects in olfactory function and the addition of CR+ PGCs, subsequent exposure to an odor-enriched environment improved these defects.

### Conclusion

The persistent subtype-specific reduction in PGCs and functional impairment after transient olfactory deprivation during the early postnatal period suggest that a critical period exists for olfactory system development similar to that of the visual system. However, extensive odor stimulation after this period resulted in efficient recovery from the histological and functional defects, which may reflect the higher plasticity of the olfactory system throughout life. These findings provide new insights into the mechanisms of postnatal neurogenesis and a biological basis for the therapeutic effect of olfactory training.

## Supporting Information

Figure S1 **Stereological quantification of PGCs at P61 in the contralateral OB of mice after 2-week neonatal olfactory deprivation and in the OB of intact mice.** There was no significant difference in the numbers of TH+, CB+, or CR+ PGCs between the contralateral OB of mice after transient occlusion (contralateral, dark blue bars, *n* = 5) and the OB of intact mice (intact, light blue bars, TH and CR: *n* = 3, CB: *n* = 4).
(TIFF)

Figure S2 **Stereological quantification of PGCs and granule cells at the end of neonatal olfactory deprivation.** The numbers of TH+, CB+, and CR+ PGCs at P19 were significantly decreased in the OB after the 2-week olfactory deprivation from P5 (occl., red bars, *n* = 4) compared with those in the control OB (control, blue bars, *n* = 4). There was no significant difference in the number of CR+ granule cells in the occluded versus control OB. GC: granule cell, *P<0.05.
(TIF)

Figure S3 **Reversible volume reduction in the OB by neonatal olfactory deprivation. A**: Nuclear staining (Hoechst 33342) images of coronal OB sections at P61 after continuous (8 w) and transient (2 w) olfactory deprivation beginning at P5. A thinner glomerular layer with small, undeveloped glomeruli was observed in the continuously occluded OB (occl. 8 w, middle), but not in the transiently occluded OB (occl. & reopening, right) compared with the control OB (control, left). **B**: Relative volume of each layer in the OB at P61. The relative volumes of the granule cell layer (GCL), external plexiform layer (EPL), and glomerular layer (GL) in the ipsilateral, occluded OB to those in the contralateral, non-occluded OB were significantly smaller in the continuous occlusion group (occl. 8 w, *n* = 4) compared with the transient occlusion group (occl. & reopening, *n* = 4). *P<0.05 Scale bar: 100 µm.
(TIFF)

Figure S4 **The ratio of the number of PGCs to the control at P19 and P61.** The ratios of the number of TH+ and CB+ PGCs in the ipsilateral OB to the contralateral (control) OB 6 weeks after naris reopening (P61) were significantly increased compared with those at the end of the occlusion (P19). On the other hand, the number of CR+ PGCs did not show such a recovery 6 weeks after plug removal. *P<0.05, **P<0.01.
(TIF)

Figure S5 **Persistent effect of transient neonatal olfactory deprivation on addition of PGCs generated after olfactory recovery. A**: Experimental procedure. Mice with transient olfactory deprivation from P5 to P19 were injected with BrdU 2 weeks after naris reopening (P33). The number of BrdU+ PGCs was quantified 6 weeks later, at P75. **B**: Quantification of the number of BrdU+ PGCs in the glomerular layer at P75. The number of BrdU+CR+ PGCs, but not of BrdU+TH+ or BrdU+CB+ PGCs was significantly smaller in the occl. & reopening group (green bars, *n* = 3) than in the control, non-occluded group (blue bars, *n* = 3). *P<0.05.
(TIFF)

Figure S6 **Neuronal progenitor quantification in V-SVZ, RMS, and OB immediately and long after neonatal olfactory deprivation.** Using coronal sections immunostained for Mash1, a neuronal progenitor marker, the number of Mash1+ cells immediately after (P19, **A,** *n* = 4) and long after (P61, **B,** *n* = 4) the two-week naris occlusion beginning at P5 were quantified. There were no significant differences in the number of Mash1+ cells in each area of the V-SVZ (M: medial, V: ventral, L: lateral, D: dorsal, C: cortical), the RMS, or the OB between the control and occluded groups at either P19 (**A**, control, blue bars; occl. 2 w, red bars) or P61 (**B**, control, blue bars; occl. & reopening, green bars).
(TIFF)

Figure S7 **Production and migration of new olfactory interneurons after neonatal olfactory deprivation. A–B**: Quantification of cell proliferation in the V-SVZ, RMS, and OB under neonatal olfactory deprivation. Mice treated with naris occlusion beginning on P5 were injected with BrdU 1 h before sacrifice at P19 (**A**). From coronal sections immunostained for BrdU, the number and distribution of BrdU+ cells in each area of the V-SVZ (M: medial, V: ventral, L: lateral, D: dorsal, C: cortical), the RMS, and each layer of the OB (core, GCL: granule cell layer, EPL: external plexiform layer, GL: glomerular layer) were determined (**B**). There was no significant difference in the number of BrdU+ cells in each area between the non-occluded (control, blue bars, *n* = 4) and occluded (occl. 2 w, red bars, *n* = 4) hemisphere. **C–D:** Distribution of BrdU-positive cells 1 week after labeling and naris plug removal. BrdU was injected followed by naris reopening at P19, and the mice were sacrificed one week later (P26) (**C**). The number and distribution of the BrdU+ cells in the V-SVZ, RMS, and each layer of the OB (core, GCL: granule cell layer, EPL: external plexiform layer, GL: glomerular layer) were examined. There was no significant difference in the number of BrdU+ cells in each area between the OBs of the non-treated (control, blue bars, *n* = 4) and reopened (occl. & reopening, green bars**,** *n* = 4) hemispheres.
(TIFF)
