# Supplementary figures and images for: A Subtype-Specific Critical Period for Neurogenesis in the Postnatal Development of Mouse Olfactory Glomeruli
Source: PLoS One. 2012 Nov 1;7(11):e48431. doi: 10.1371/journal.pone.0048431 (PMC3486849; doi:10.1371/journal.pone.0048431)

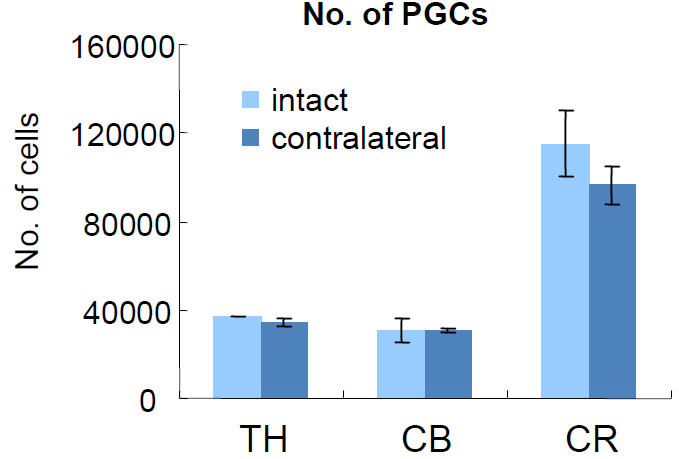

Supplement: Figure S1 — Stereological quantification of PGCs at P61 in the contralateral OB of mice after 2-week neonatal olfactory deprivation and in the OB of intact mice. There was no significant difference in the numbers of TH+, CB+, or CR+ PGCs between the contralateral OB of mice after transient occlusion (contralateral, dark blue bars, n = 5) and the OB of intact mice (intact, light blue bars, TH and CR: n = 3, CB: n = 4). (TIFF) [file pone.0048431.s001.tiff]

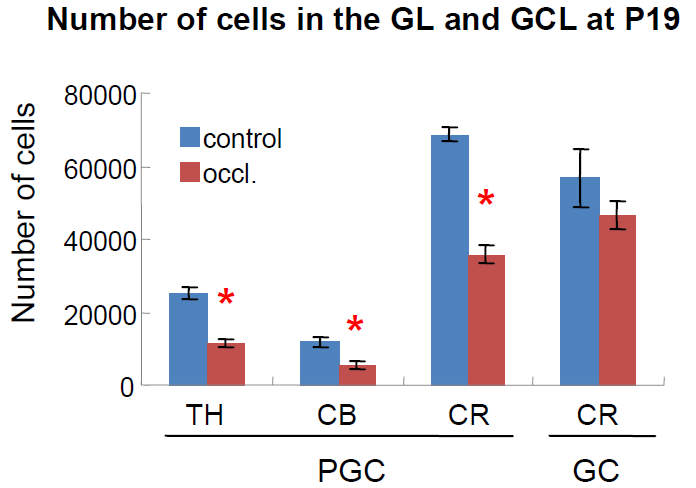

Supplement: Figure S2 — Stereological quantification of PGCs and granule cells at the end of neonatal olfactory deprivation. The numbers of TH+, CB+, and CR+ PGCs at P19 were significantly decreased in the OB after the 2-week olfactory deprivation from P5 (occl., red bars, n = 4) compared with those in the control OB (control, blue bars, n = 4). There was no significant difference in the number of CR+ granule cells in the occluded versus control OB. GC: granule cell, *P<0.05. (TIF) [file pone.0048431.s002.tif]

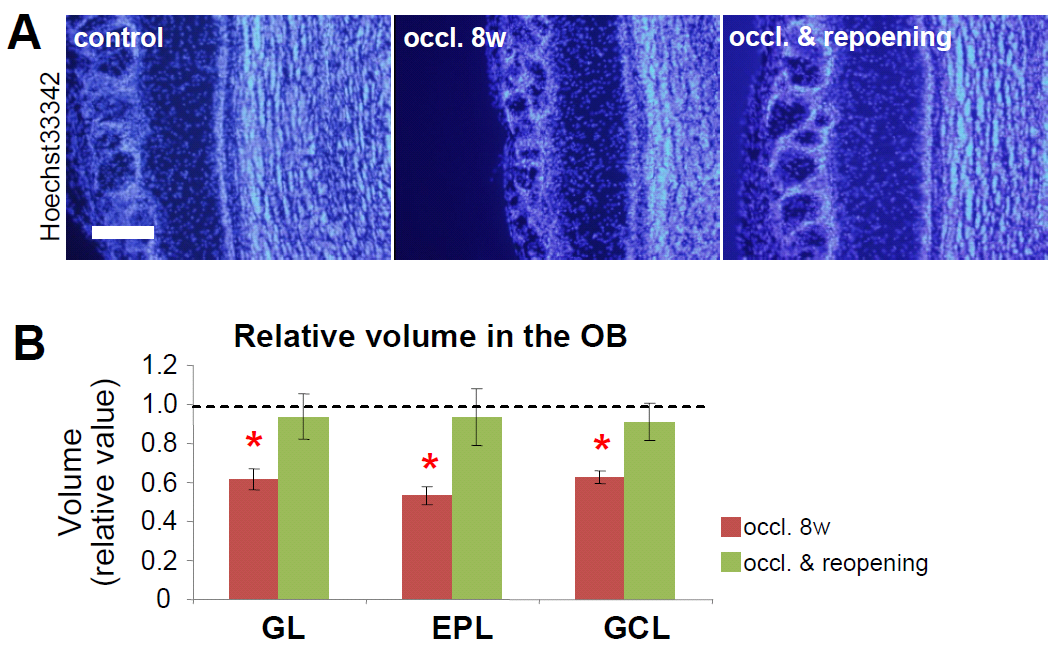

Supplement: Figure S3 — Reversible volume reduction in the OB by neonatal olfactory deprivation. A: Nuclear staining (Hoechst 33342) images of coronal OB sections at P61 after continuous (8 w) and transient (2 w) olfactory deprivation beginning at P5. A thinner glomerular layer with small, undeveloped glomeruli was observed in the continuously occluded OB (occl. 8 w, middle), but not in the transiently occluded OB (occl. & reopening, right) compared with the control OB (control, left). B: Relative volume of each layer in the OB at P61. The relative volumes of the granule cell layer (GCL), external plexiform layer (EPL), and glomerular layer (GL) in the ipsilateral, occluded OB to those in the contralateral, non-occluded OB were significantly smaller in the continuous occlusion group (occl. 8 w, n = 4) compared with the transient occlusion group (occl. & reopening, n = 4). *P<0.05 Scale bar: 100 µm. (TIFF) [file pone.0048431.s003.tiff]

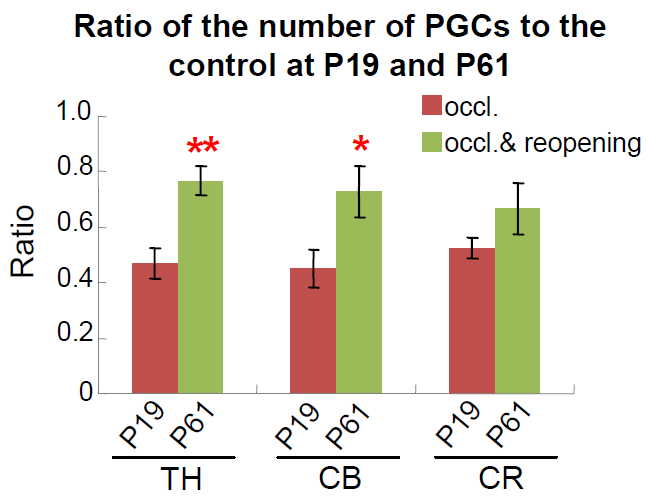

Supplement: Figure S4 — The ratio of the number of PGCs to the control at P19 and P61. The ratios of the number of TH+ and CB+ PGCs in the ipsilateral OB to the contralateral (control) OB 6 weeks after naris reopening (P61) were significantly increased compared with those at the end of the occlusion (P19). On the other hand, the number of CR+ PGCs did not show such a recovery 6 weeks after plug removal. *P<0.05, **P<0.01. (TIF) [file pone.0048431.s004.tiff]

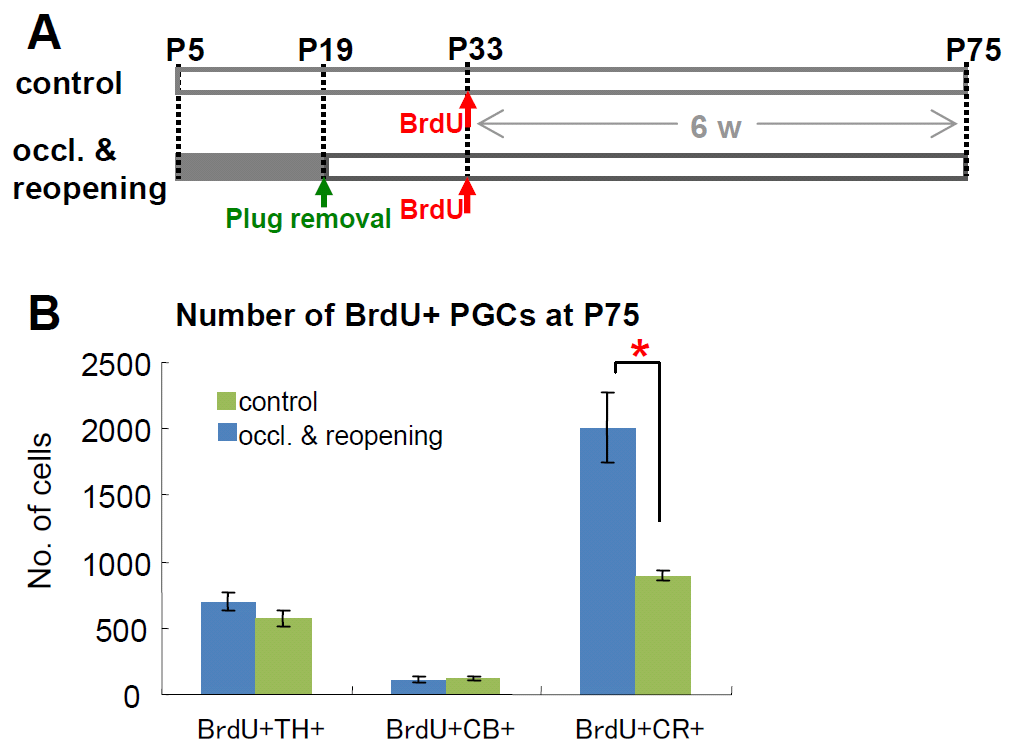

Supplement: Figure S5 — Persistent effect of transient neonatal olfactory deprivation on addition of PGCs generated after olfactory recovery. A: Experimental procedure. Mice with transient olfactory deprivation from P5 to P19 were injected with BrdU 2 weeks after naris reopening (P33). The number of BrdU+ PGCs was quantified 6 weeks later, at P75. B: Quantification of the number of BrdU+ PGCs in the glomerular layer at P75. The number of BrdU+CR+ PGCs, but not of BrdU+TH+ or BrdU+CB+ PGCs was significantly smaller in the occl. & reopening group (green bars, n = 3) than in the control, non-occluded group (blue bars, n = 3). *P<0.05. (TIFF) [file pone.0048431.s005.tiff]

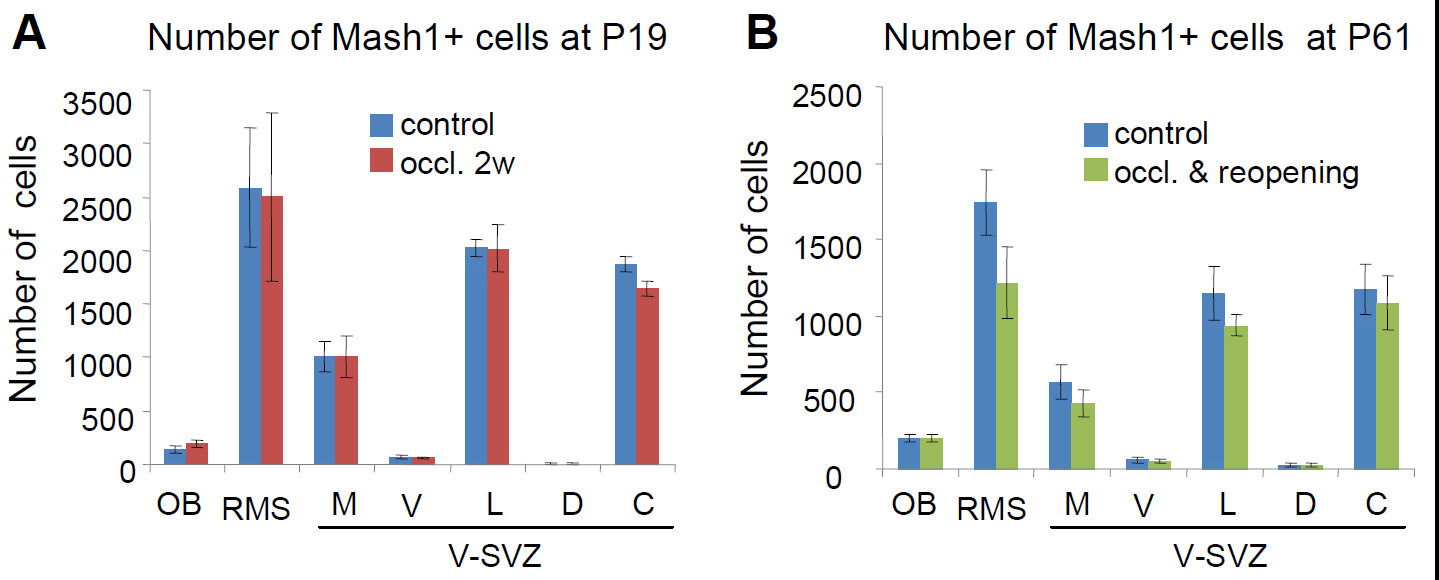

Supplement: Figure S6 — Neuronal progenitor quantification in V-SVZ, RMS, and OB immediately and long after neonatal olfactory deprivation. Using coronal sections immunostained for Mash1, a neuronal progenitor marker, the number of Mash1+ cells immediately after (P19, A, n = 4) and long after (P61, B, n = 4) the two-week naris occlusion beginning at P5 were quantified. There were no significant differences in the number of Mash1+ cells in each area of the V-SVZ (M: medial, V: ventral, L: lateral, D: dorsal, C: cortical), the RMS, or the OB between the control and occluded groups at either P19 (A, control, blue bars; occl. 2 w, red bars) or P61 (B, control, blue bars; occl. & reopening, green bars). (TIFF) [file pone.0048431.s006.tiff]

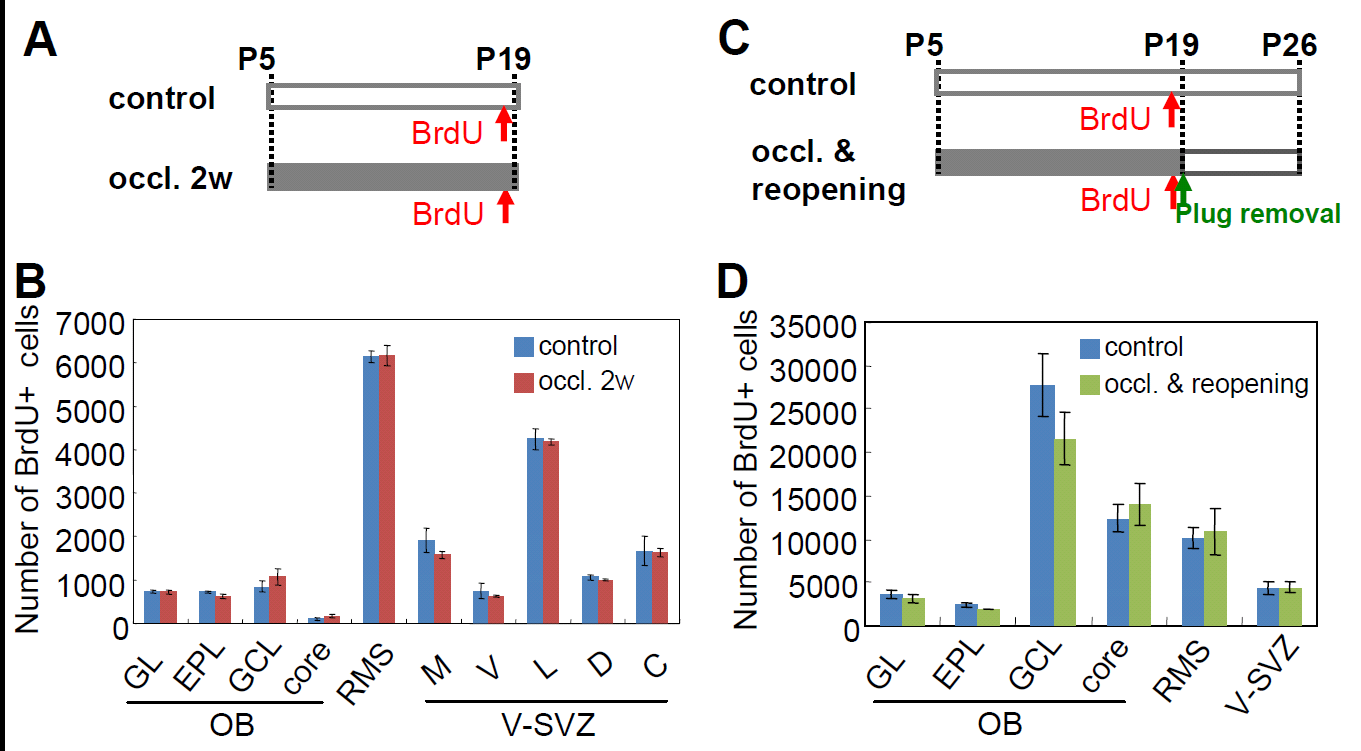

Supplement: Figure S7 — Production and migration of new olfactory interneurons after neonatal olfactory deprivation. A–B: Quantification of cell proliferation in the V-SVZ, RMS, and OB under neonatal olfactory deprivation. Mice treated with naris occlusion beginning on P5 were injected with BrdU 1 h before sacrifice at P19 (A). From coronal sections immunostained for BrdU, the number and distribution of BrdU+ cells in each area of the V-SVZ (M: medial, V: ventral, L: lateral, D: dorsal, C: cortical), the RMS, and each layer of the OB (core, GCL: granule cell layer, EPL: external plexiform layer, GL: glomerular layer) were determined (B). There was no significant difference in the number of BrdU+ cells in each area between the non-occluded (control, blue bars, n = 4) and occluded (occl. 2 w, red bars, n = 4) hemisphere. C–D: Distribution of BrdU-positive cells 1 week after labeling and naris plug removal. BrdU was injected followed by naris reopening at P19, and the mice were sacrificed one week later (P26) (C). The number and distribution of the BrdU+ cells in the V-SVZ, RMS, and each layer of the OB (core, GCL: granule cell layer, EPL: external plexiform layer, GL: glomerular layer) were examined. There was no significant difference in the number of BrdU+ cells in each area between the OBs of the non-treated (control, blue bars, n = 4) and reopened (occl. & reopening, green bars, n = 4) hemispheres. (TIFF) [file pone.0048431.s007.tiff]
